# Basal luteinizing hormone and follicular stimulating hormone: is it sufficient for diagnosis of precocious puberty in Korean girls?

**DOI:** 10.1186/1687-9856-2013-S1-P72

**Published:** 2013-10-03

**Authors:** Sun Hee Lee, Woo Yeung Chung, Jae Hyun Kim

**Affiliations:** 1 Inje University College of Medicine, Department of Pediatrics, Busan Paik Hospital, Busan, Korea; 2 Inje University College of Medicine, Department of Pediatrics, Ilsan Paik Hospital, Goyang, Korea

## Aims

A GnRH stimulation test is a gold standard in diagnosing central precocious puberty. The aim of this study was to evaluate the auxological and biochemical characteristics of Korean girls who had undergone GnRH stimulation test.

## Methods

In 2 tertiary pediatric centers, Korean girls with early pubertal development were included during 2010-2011. Height, BMI, pubertal stage, chronological age and bone agewere evaluated. LH, FSH, estradiol, IGF-I and IGFBP-3 were measured and a standard GnRH stimulation test was performed.The patients with peripheral precocious puberty were excluded. The peak LH level of ≥5 IU/L was considered pubertal response during GnRH tests.

## Results

Among 302 girls had undergone GnRH stimulation tests, 299 girls were included. Three girls were excluded with peripheral precocious puberty due to ovarian cysts. The chronological age of 299girls was 8.23(0.68) yr and bone age was 9.90(0.96) yr at the time of a GnRH test. The pubertal responses were observed in 226 girls (75.6%), and prepubertal responses were in 73girls (24.4%). There were significant differences in basal LH, peak LH, basal FSH, peak LH/FSH ratio, estradiol and BMI SDS between two groups. In logistic regression analysis, odds ratio of basal LH was 24.30 (95% C.I. 2.14-275.73, P=0.01). Receiver operating characteric analysis showed that basal LH/FSH ratio is a better predicting method for the pubertal result after GnRH stimulation test over basal LH, FSH and estradiol (area under the curve was 0.948, 0.757, 0.714 and 0.565, respectively). Among 150 girls with a basal LH of <0.1 IU/L, 89 (59.3%) had pubertal responses. There was no predicting factor in logistic regression analysis in girls with a basal LH below detection limit.

## Conclusion

An elevated level of basal LH/FSH ratio was a significant predicting factor in pubertal responses during GnRH stimulation tests. However activation of HPG axis was observed even in girls with a basal LH below detection limit.A GnRH stimulation test was still necessary for a diagnostic confirmation of central precocious puberty.

